# The role of hRev7, the accessory subunit of hPolζ, in translesion synthesis past DNA damage induced by benzo[a]pyrene diol epoxide (BPDE)

**DOI:** 10.1186/1471-2121-11-97

**Published:** 2010-12-10

**Authors:** Jessica A Neal, Kathryn L Fletcher, J Justin McCormick, Veronica M Maher

**Affiliations:** 1 Carcinogenesis Laboratory, Department of Microbiology & Molecular Genetics, and Department of Biochemistry & Molecular Biology, Michigan State University, East Lansing, MI 48824-1302, USA

## Abstract

**Background:**

DNA polymerase zeta (Polζ) is a specialized DNA polymerase that, unlike classical replicative polymerases, is capable of replicating past DNA lesions, i.e. of performing translesion synthesis (TLS). The catalytic subunit of hPolζ, hRev3, has been shown to play a critical role in DNA damage-induced mutagenesis in human cells, but less is known about the role of hRev7, the accessory subunit of hPolζ, in such mutagenesis. To address this question, we recently generated human fibroblasts with very significantly reduced levels of hRev7 protein and demonstrated that hRev7 is required to protect cells from ultraviolet_(254 nm) _(UV) radiation-induced cytotoxicity and mutagenesis (McNally et al., DNA Repair 7 (2008) 597-604). The goal of the present study was to determine whether hRev7 is similarly involved in the tolerance of DNA damage induced by benzo[a]pyrene diol epoxide (BPDE), the reactive form of the widespread environmental carcinogen benzo[a]pyrene.

**Methods:**

To determine whether hRev7 also plays a role in protecting human cells from the cytotoxicity and mutagenesis induced by benzo[a]pyrene diol epoxide (BPDE), cell strains with reduced hRev7 were compared to their parental strain and a vector control strain for the effect of BPDE on cell survival, induction of mutations, and the ability to progress through the cell cycle.

**Results:**

The results show that cell strains with reduced hRev7 are more sensitive to the cytotoxic effect of BPDE than the control strains, and progress through S-phase at a slower rate than the control cells following BPDE treatment, indicating that hRev7, and likely hPolζ, is required for efficient bypass of BPDE-induced DNA lesions. However, neither the frequency nor kinds of mutations induced by BPDE in cells with reduced hRev7 differ significantly from those induced in the control strains, suggesting that hPolζ is not essential for inserting nucleotides opposite BPDE-induced DNA damage.

**Conclusions:**

Taken together, our results which show that hRev7 is required for TLS past BPDE-induced DNA lesions but that it is not essential for inserting nucleotides opposite such lesions suggest a role for hPolζ in the extension step of translesion synthesis.

## Background

Human cells undergo countless rounds of DNA replication, which must be very accurate to preserve critical genetic information. To maintain such a significant level of accuracy, the classical replicative polymerases have evolved highly selective active sites that only accommodate nucleotides when they are correctly paired to the DNA template. In addition, many of these DNA polymerases possess 3'→5' proofreading exonuclease activity, which removes nucleotides that are incorrectly incorporated during replication, allowing an additional attempt at accurate DNA synthesis. Because of their stringency, the classical replicative polymerases cannot tolerate fluctuations in the DNA structure, including those that result from DNA damage. Nevertheless, DNA is continually subjected to a variety of insults, from both endogenous and environmental agents, that generate DNA damage. Much of this damage is excised by DNA repair mechanisms before replication occurs. However, if repair is slow or the DNA damage is extensive, DNA lesions may persist during replication. If the high fidelity replicative polymerase complex encounters a DNA lesion that blocks elongation, potentially fatal stalling or arrest of replication can occur.

To avoid replication arrest, mechanisms have evolved that enable DNA lesions to be tolerated without their physical removal. Translesion synthesis (TLS) is one such mechanism. Translesion synthesis involves the use of specialized polymerases, that are thought to bypass DNA lesions using a two-step mechanism where, nucleotides are first inserted opposite DNA damage and then the resulting atypical primer termini are extended, before the replicative polymerases resume DNA synthesis (For review see [1]). Several DNA polymerases have been discovered, whose primary function appears to be TLS. These TLS polymerases typically contain active sites that are less restrictive, making them able to accommodate distortions in DNA (see for example [2-5]). Although TLS polymerases have the unique ability to synthesize past replication-blocking DNA lesions, enabling cells to survive such DNA damage, they are also characterized by relaxed nucleotide selectivity and lack of 3'→5' proofreading exonuclease activity. As a result, protection of cells from replication arrest may come at the cost of introducing mutations in DNA, which can contribute to the development of cancer.

More than 300 polymerases involved in TLS have been discovered in eukaryotes, bacteria and archaea [6]. The first TLS polymerase to be identified in eukaryotes was DNA polymerase zeta (Polζ) [7]. DNA polymerase ζ was initially characterized in the budding yeast, *Saccharomyces cerevisiae*, and is composed of two subunits, a catalytic subunit, called Rev3, as well as an accessory subunit, Rev7 [7]. Studies using yeast *rev *mutant strains have demonstrated that Polζ is responsible for the majority of both spontaneous [8,9] and DNA damage-induced mutations that occur in this organism [10-15], suggesting that this polymerase participates in error-prone TLS past an extensive array of DNA lesions (reviewed in [16]).

Human homologs of the yeast *REV *genes have been identified [17,18]. The transcript of the human *REV3 *gene encodes a 353 kDa protein, which is about twice the size of the yeast protein [19]. Presumably because of the large size and low cellular levels of hRev3, the protein has never been expressed or isolated and therefore, *in vitro *studies using human Polζ are lacking [20]. However, human cells expressing high levels of *hREV3 *antisense RNA have been reported to demonstrate a lower frequency of ultraviolet (UV)-induced mutations than the control cells, indicating that, as in yeast, hRev3 is required for induced mutagenesis and suggesting that the functions of Polζ are conserved from yeast to humans [19,21].

To investigate the role of hRev7 in TLS, we recently generated two human fibroblast cell strains in which the levels of hRev7 protein were significantly reduced by small interfering RNA (siRNA) [22]. When cell strains with reduced hRev7 were UV-irradiated, their rate of progression through S-phase was considerably slower, and their cell survival was significantly reduced, compared to control strains. In addition, the frequency of UV-induced mutations in cell strains with reduced hRev7 was five times lower than normal. These data show that like hRev3, hRev7, presumably as a part of human Polζ, plays a role in UV-induced mutagenesis of human cells.

To determine whether hRev7 is similarly involved in the tolerance of DNA damage induced by benzo[a]pyrene diol epoxide (BPDE), the reactive form of the widespread environmental carcinogen benzo[a]pyrene, cells strains with reduced levels of hRev7 were compared to their parental strain and a vector control for their response to the biological effects of BPDE. Our results show that cell strains with reduced hRev7 progress through the cell cycle at a slower rate than control strains after exposure to BPDE, and are also more sensitive to its cytotoxic effect. These data suggest that in the absence of hRev7, cells are less efficient at completing TLS past BPDE-induced DNA lesions, resulting in a delay in cell cycle progression and increased cell death following exposure to BPDE. To our surprise, however, we found that neither the frequency nor the kinds of mutations induced by BPDE in cells with reduced levels of hRev7 differ dramatically from those induced in the control cell strains, suggesting that hRev7 is not responsible for the insertion of nucleotides opposite BPDE-induced DNA lesions. Together, these results are consistent with a role for hRev7, and likely hPolζ, in the extension step of TLS past BPDE-induced DNA lesions.

## Methods

### Cell strains

The human fibroblast cell strain used as the parental strain in this study, designated MSU-1.2.9N.58 (9N.58 for short), was derived from the infinite life span, telomerase positive, near-diploid, karyotypically stable, MSU-1.2 lineage of cells established in the Carcinogenesis Laboratory [23]. Cell strains 2-2 and 2-6, which have significantly reduced levels of hRev7 protein, as well as the vector control strain, VCA, were derived from the parental strain, 9N.58, by McNally et al., as described [22]. The two additional cell strains with reduced hRev7 that were used in this study, designated 2.5 and 3.2, as well as the vector control, V1.1, were derived from the same parental strain, i.e. 9N.58, using the methods previously described [22]. To generate a cell strain in which the level of hRev7 has been reconstituted (2-2 + R7), cell strain 2-2 was transfected with a vector that expresses an siRNA insensitive hRev7 mRNA using Lipofectamine (Invitrogen) according to the manufacturer's instructions.

### Western blot analysis

Nuclear protein extracts were obtained and Western analysis was conducted as previously described [22].

### Exposure of cells to chemical mutagens

When treating with chemical mutagens, the number of DNA lesions generated is dependent upon the cell density at the time of treatment. Therefore, both for survival and for mutagenesis studies, cells in exponential growth were detached from the dishes using trypsin and plated in 150 mm-diameter dishes approximately 16 h prior to treatment, such that the density of cells at the time of treatment would be as near 10,000 cells/cm^2 ^as possible. Following the 16 h attachment period, the culture medium was removed from each dish, cells were rinsed twice with phosphate-buffered saline (PBS), and then covered in Eagle's minimal essential medium [for MNU treatments, medium was buffered with 15 mM HEPES (pH 7.2)]. Immediately prior to treatment, BPDE (Midwest Research Institute) or MNU (Sigma) were dissolved in anhydrous dimethylsulfoxide (DMSO), and the designated doses were delivered by micropipette. To ensure that all cells were exposed to the same concentration of DMSO, regardless of the dose of BPDE or MNU, appropriate amounts of DMSO were added to dishes (including the untreated control cells) to equal the total amount of DMSO delivered to cells treated with the highest dose. Cisplatin (American Pharmaceutical Partners Inc.), which was supplied in an aqueous saline solution (1 mg/ml), was delivered to the dishes directly by micropipette. Cells were exposed to BPDE or cisplatin for 1 h or to MNU for 30 min, at 37°C in a humidified 5% CO_2 _incubator. At the end of the exposure period the medium containing the mutagen was removed, cells were rinsed twice with PBS, and supplied with fresh culture medium. Induced cytotoxicity and mutagenesis assays were performed as described below.

### Exposure of cells to ionizing radiation

On the day of treatment, exponentially growing cells were detached from dishes with trypsin and diluted to 200,000 cells/ml in culture medium containing 2% supplemented calf serum. Cells were irradiated as described [24] in 50 ml polypropylene tubes on ice using a U.S. Nuclear ^60^Co variable flux, sealed source irradiator with a dose rate of 1.378 Gy/min.

### Cell survival assay

The procedures for determining the cytotoxic effects of DNA damaging agents by colony forming ability differ slightly based upon the particular type of DNA damaging agent used. For chemical mutagens, the cells were exposed to the appropriate agent at a density of 10,000 cells/cm^2 ^as described above. Immediately following treatment, the cells were rinsed with PBS, and detached from the dishes using trypsin. Cells were then diluted and plated into four 100 mm-diameter dishes for each dose at cloning densities (i.e. the densities necessary to obtain approximately 50 surviving colonies per 100 mm-diameter dish depending on the expected cytotoxicity). After 7 days, cells were provided with fresh culture medium, and after 14 days, they were stained with crystal violet. To determine sensitivity to the cytotoxic effect of a particular agent (expressed as percent survival), the cloning efficiencies of cells exposed to the mutagen were normalized to the cloning efficiency of the untreated control cells.

To determine the cytotoxicity induced by ionizing radiation, cells were detached using trypsin and irradiated in suspension as described above. Immediately after irradiation, each cell suspension was diluted appropriately into fresh culture medium and plated into 4, 100 mm-diameter dishes for each dose at cloning densities. The culture medium was renewed after 7 days and the cells were stained after 14 days. The survival of the irradiated cells was calculated by normalizing the cloning efficiency of the irradiated cells to that of the control cells.

### Mutagenesis assay

To determine the frequency of induced mutations in the *hypoxanthine phosphoribosyl transferase (HPRT) *gene, assays were performed as described [25]. In short, a sufficient number of target cells were plated into 150 mm-diameter dishes to ensure that after treatment, the number of surviving cells was large enough to result in at least 40, 6-thioguanine (TG) resistant clones. Because exposure to BPDE causes a high frequency of induced mutants, it is sufficient to have approximately 0.8 × 10^6 ^surviving cells. Following exposure to BPDE, cells were maintained in exponential growth for an 8-day expression period to allow any wild-type HPRT protein to be depleted. After the 8-day expression period, cells were trypsinized and diluted to 2,500 cells/ml in culture medium. To determine the cloning efficiency of the cells at the time of selection, a small portion of the cell suspension was diluted further and plated into 4, 100 mm-diameter dishes at cloning densities. To assay for TG-resistance, the remainder of the cell suspension was selected with TG, at a final concentration of 40 μM, and then cells were plated at a density of 25,000 cells per 100 mm-diameter dish. All dishes were supplied with fresh culture medium (with or without TG as appropriate) after 7 days, and stained with crystal violet after 14 days. The observed mutation frequency was corrected by the cloning efficiency of the unselected cells. The induced mutation frequency was calculated by subtracting corrected frequencies observed in untreated control cells from the corrected mutant frequencies of the treated cells.

### Analysis of the types of base substitutions induced by BPDE in the *HPRT *gene

To determine the kinds of mutations induced in the *HPRT *gene, TG-resistant clones obtained from doses of BPDE that resulted in mutation frequencies more than ten times the background frequency, were isolated, and then lysed, and their *HPRT *cDNA was amplified as described previously [22]. Only base substitutions that occurred at adenine or guanine and resulted in an amino acid change were considered to be BPDE-induced. When two clones were considered to be siblings, i.e. they contained the same mutation, only one of the mutants was included in the study.

### Analysis of the cell cycle progression of BPDE treated cells by flow cytometry

Cells in exponential growth were detached from the dishes using trypsin, and plated in 100 mm-diameter dishes such that the density of G_1_/S-synchronized cells at the time of treatment would be 10,000 cells/cm^2^. Cells were allowed 16 h to attach and then synchronized at the G_1_/S border exactly as described [22]. Cells were released from synchrony by washing twice with PBS and immediately treated with BPDE as described above. Every 4 h for the first 24 h post-treatment, cells were fixed in 80% ethanol and stained with a propidium iodide solution as described previously [22]. Cells were analyzed for DNA content by flow cytometry at the Flow Cytometry Core Facility at Michigan State University.

## Results

### Effect of reduced hRev7 protein on the survival of human fibroblasts exposed to BPDE and on the frequency of BPDE-induced mutations

**Figure 1:**
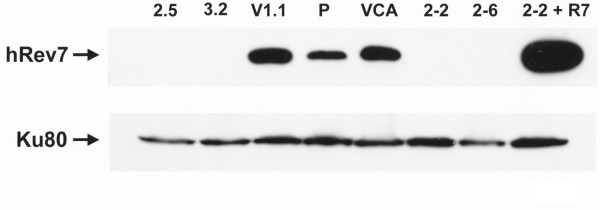
**Western blot analysis of the level of hRev7 protein**. The level of hRev7 protein was examined by Western blotting of nuclear extracts obtained from the parental cell strain (P), derivative strains expressing hRev7 siRNA (clones 2-2, 2-6, 2.5 and 3.2), control strains expressing an siRNA with limited homology to known sequences in the human genome (VCA and V1.1), as well as a complementation derivative of cell strain 2-2 that expresses an siRNA insensitive hRev7 (2-2 + R7). Ku80 was used as a loading control.

Using siRNA, we previously generated two derivatives of the human fibroblast strain 9N.58 with significantly reduced levels of hRev7 protein [22]. These derivative strains were designated 2-2 and 2-6. Those two cell strains, along with two newly generated 9N.58-derived cell strains with reduced hRev7 protein (designated 2.5 and 3.2), as well as the appropriate vector controls (VCA and V1.1), were used in our current study (Figure 1). In addition, the level of hRev7 was also reconstituted in cell strain 2-2 (2-2 + R7) by transfecting that cell strain with a vector expressing hRev7 mRNA with a nucleotide sequence that was altered in such a way as to make it insensitive to siRNA-induced degradation (Figure 1).

**Figure 2:**
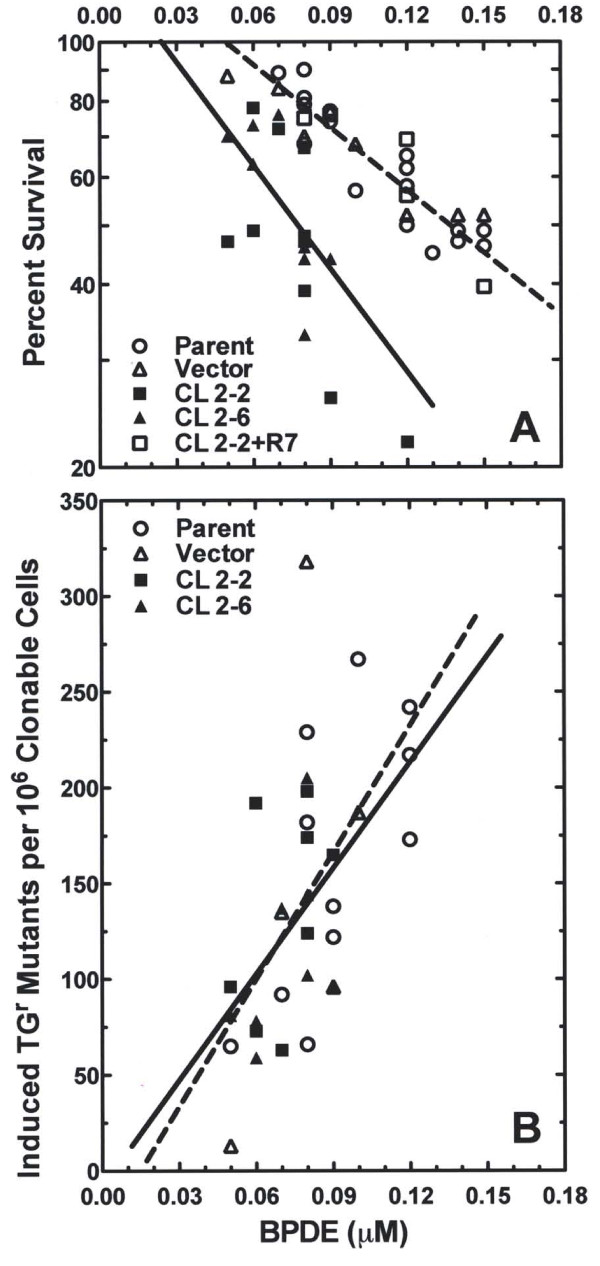
**Reducing the level of hRev7 in human fibroblasts renders them more sensitive to the cytotoxic effect of BPDE, but does not affect their frequency of BPDE-induced mutations**. The parental cells strain (open circles); cell strains with reduced hRev7, designated clones 2-2 and 2-6 (closed symbols); the vector control strain (open triangles); and a cell strain in which to level of hRev7 was reconstituted, designated 2-2 + R7 (open squares); were treated with BPDE and assayed for (A) cell survival or (B) the frequency of mutations induced in the *HPRT *gene. The lines represent the least squares regression for the data.

To determine whether reducing the level of hRev7 protein alters the response of human fibroblast cells to the cytotoxic or mutagenic effects of BPDE, two cell strains with significantly reduced levels of hRev7 protein, (clones 2-2 and 2-6) were assayed along with their parental strain and a vector control transfectant for their sensitivity to the cytotoxic effect of BPDE as measured by survival of colony-forming ability (Figure 2A). These data show that, whereas the vector control strain demonstrated a BPDE-induced cytotoxicity that was very similar to that of the parent strain, each of the cell strains with reduced hRev7 protein was considerably more sensitive to BPDE-induced cytotoxicity. Specifically, 80% of the parent and vector control cells survived after being exposed to 0.07 μM BPDE, but only 50% of cells with reduced hRev7 survived following exposure to the same dose. Complementation of cell strain 2-2 with hRev7 (2-2 + R7) resulted in BPDE-induced sensitivities that were indistinguishable from those of the control cell stains, indicating that the marked increase in sensitivity to the cytotoxic effect of BPDE demonstrated by cells with reduced hRev7 was not the result of an unintended, off-target effect of the siRNA. Together, these results show that cells with reduced hRev7 are more sensitive to the cytotoxic effect of BPDE than control cells.

Because cell strains with reduced hRev7 protein were more sensitive to the cytotoxic effect of BPDE, we were interested to determine whether the increase in the killing of such cells occurred as a result of a requirement for this protein in TLS past BPDE-induced DNA lesions, as was found with UV [22]. Therefore, cell strains with reduced hRev7 were compared to their parental cell strain as well as to the vector control strain for the frequency of mutations induced in the *HPRT *gene following exposure to BPDE. To our surprise, the BPDE-induced mutation frequencies of cell strains with reduced expression of hRev7 protein did not differ significantly from those of their parental cell strain or from those of the vector control cell strain (Figure 2B).

### Effect of reduced hRev7 protein on the types of BPDE-induced base substitutions in human fibroblasts

Although we were unable to detect any differences between the frequency of mutations induced by BPDE in cell strains with reduced hRev7 and control strains, it is possible that, in the absence of hPolζ, another TLS polymerase, which makes mutations at a very similar frequency to hPolζ, substitutes. However, it is unlikely that another TLS polymerase would make the same frequency and types of mutations as hPolζ does. Therefore, we also compared the kinds of mutations induced by BPDE in cell strains with reduced hRev7 and control strains, by isolating BPDE-induced, TG-resistant colonies and sequencing their DNA for mutations in the *HPRT *gene. The results are compared in Table 1. Although the percentage of G→C mutations induced by BPDE in control cells differs somewhat from those in cell strains with reduced hRev7, based on the Fisher Exact Test, the frequency 8 out of 45 is not statistically different from 2 out of 37 (one-sided p-value = 0.084). Thus, these data indicate that the kinds of base substitutions induced by BPDE in cells with reduced hRev7 do not differ from those induced in normal human fibroblasts.

**Table 1:** Types of base substitutions induced by BPDE in the *HPRT* gene of cells with normal or reduced levels of hRev7

Base changes	Parent and vector control	Clone 2-2 and clone 2-6
G→T	29 (64.4%)	28 (75.7%)
G→A	2 (4.4%)	4 (10.8%)
G→C	8 (17.8%)	2 (5.4%)
A→T	1 (2.2%)	0 (0%)
A→G	3 (6.7%)	0 (0%)
A→C	2 (4.4%)	3 (8.1%)

Total	45 (100%)	37 (100%)

### Effect of reduced hRev7 on cell cycle progression following BPDE treatment

Our laboratory previously demonstrated that decreasing the level of hRev7 protein in human fibroblasts rendered them more sensitive to the cytotoxic effect of UV and resulted in impaired progression through S-phase following UV-irradiation [22]. We hypothesized that this UV-induced delay in cell cycle progression contributed to the increased UV-induced cytotoxicity that we observed. In the present study we found that, as with UV, cell strains with reduced hRev7 are more sensitive to the cytotoxic effect of BPDE than control strains. Therefore, we examined the ability of these cell strains to progress through the cell cycle following BPDE treatment.

**Figure 3:**
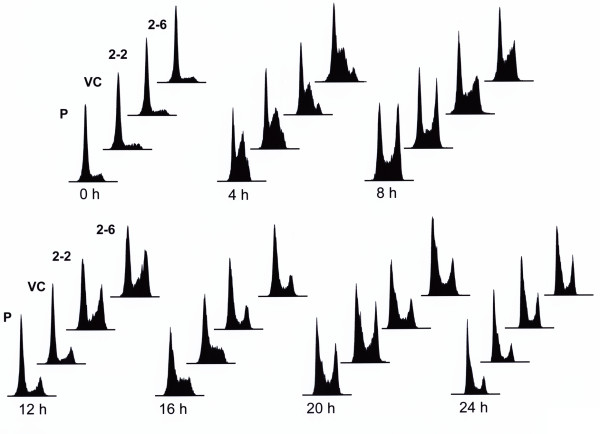
**Reducing the level of hRev7 in human fibroblasts results in a BPDE-induced delay in progression through the cell cycle**. Cell strains with reduced levels of hRev7 (2-2 and 2-6), their parental cell strain (P), and a vector control transfectant (VC) were synchronized at the G1/S border and treated with BPDE for one h immediately after release from synchrony. Cells were harvested, fixed, and stained with propidium iodide for analysis of DNA content by flow cytometry. The distribution of cells in each phase in the cell cycle 0 h, 4 h, 8 h, 12 h, 16 h, 20 h, and 24 h post-BPDE treatment are depicted.

To determine the effect of decreased expression of hRev7 protein on the rate of cell cycle progression of BPDE-treated cells, the cell strains 2-2 and 2-6, with reduced hRev7, their parental cell strain (P) and the vector control strain (VC) were synchronized at the G_1_/S border, released from synchrony, and exposed to BPDE for 1 h. At the end of BPDE exposure, populations of each of the four cell strains were harvested every 4 h for 24 h and analyzed by flow cytometry to determine the percentage of cells in each stage of the cell cycle. The resulting DNA histograms obtained from the sets of cells harvested at various times after BPDE treatment are shown in Figure 3. For each of the cell strains, immediately after release from synchrony and just prior to BPDE treatment (0 h), the majority cells were located at the G_1_/S border, indicating that each of the four cell strains synchronized equally well. After 4 h, most of the cells previously synchronized at the G_1_/S border had progressed into S-phase regardless of the level of hRev7 protein, indicating that cells released from the block and entered S-phase at similar rates post-BPDE treatment. Eight h after BPDE treatment, many of the synchronized control cells had moved through S-phase and entered into the G_2 _phase, whereas fewer of the cells with reduced hRev7 had completed S-phase, indicating that following BPDE treatment, cell strains with reduced hRev7 progressed through S-phase at a slower rate than the two control strains. After 12 h, most of the control cells had divided and cycled back into G_1_. In contrast, cells with reduced hRev7 were still primarily in S and G_2 _phase. Sixteen h post-BPDE treatment, the control strains had progressed through G_1 _and back into S-phase, whereas the cell strains with reduced hRev7 were primarily in G_2 _and G_1_, a distribution similar to that of the control cells 4 h earlier. After 20 h, although only a proportion of the cells remained synchronized, the synchronized control cells were moving through S-phase and into G_2_, but the cells with reduced hRev7 were delayed in moving into S phase and G_2 _phase, indicating that, even after entering a second cell cycle, cells with reduced hRev7 progressed somewhat slower than the control cells. Twenty-four h post-BPDE treatment, the synchrony of all four cell strains was lost. Taken together, the data presented in Figure 3 indicate that, after BPDE treatment, cells with reduced hRev7 progress more slowly through the cell cycle than the control strains.

### Effect of reduced hRev7 on the survival of cells exposed to DNA damaging agents

**Figure 4:**
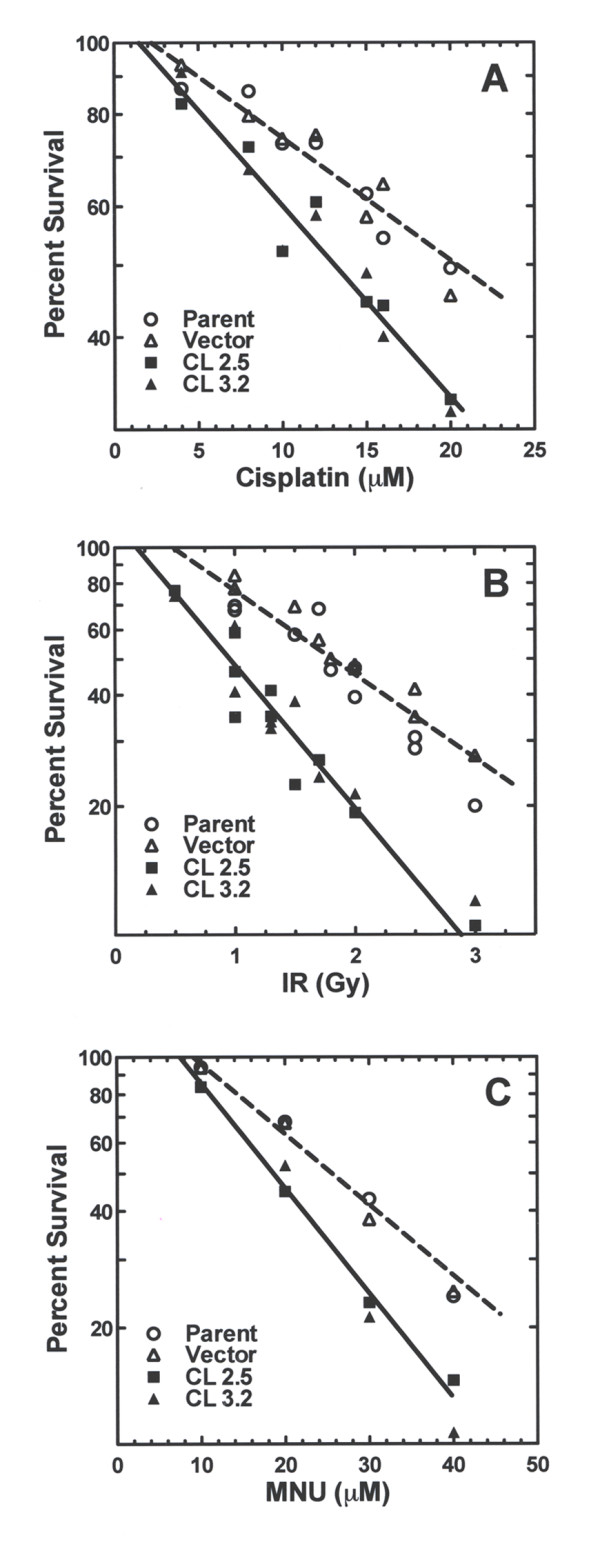
**Reducing the level of hRev7 in human fibroblast cells renders them more sensitive to the cytotoxic effects of a variety of different types of DNA damaging agents**. Cell strains 2.5 and 3.2, which have significantly reduced hRev7 protein, (closed symbols) were compared to their parental strain and the vector control strain (open symbols) for their sensitivity to the cytotoxic effects of (A) the DNA cross-linking agent cisplatin, (B) ionizing radiation (IR), and (C) the alkylating agent *N*-methyl-*N*-hydroxyurea (MNU). The lines represent the least squares regression for the data.

Cell strains with reduced hRev7 are sensitive to the cytotoxic effects of both UV and BPDE, which generate structurally distinct types of DNA lesions. Therefore, we examined whether the cell strains with reduced hRev7 also differed from the control strains in their response to the cytotoxic effects of other types of DNA lesions by exposing them to ionizing radiation; the DNA cross-linking agent, cisplatin; and the alkylating agent, MNU. As shown in Figure 4, cell strains with reduced hRev7 were more sensitive to cell killing induced by each of these three DNA damaging agents, suggesting that hRev7 is required for TLS past a variety of distinct types of DNA damage.

## Discussion

The results presented show that reducing the level of hRev7 in human fibroblasts has no significant effect on the frequency (Figure 2B) or kinds (Table 1) of mutations induced by BPDE in such cell strains, suggesting that hRev7 is not required for inserting nucleotides opposite DNA lesions induced by this damaging agent. However, because our data indicate a requirement for hRev7 in survival (Figure 2A) and cell cycle progression (Figure 3) following exposure to BPDE, these data suggest that hRev7 is required for efficient TLS past BPDE-induced DNA lesions.

Using a gapped plasmid assay, Shachar et al. [26] have recently demonstrated that at least 39% of BPDE adducts were bypassed by a TLS pathway that requires the combined actions of Polζ and Polκ. They suggest that such adducts are bypassed using a two-step, two-polymerase mechanism whereby Polκ first inserts a nucleotide opposite a BPDE-induced DNA lesion and then Polζ performs the subsequent extension step. Our study is consistent with such a mechanism. The fact that there are no significant differences between the frequency or kinds of mutations induced by BPDE in cells, regardless of the level of hRev7 expression, indicates that a polymerase other than Polζ (possibly Polκ or Polη) is required for the insertion step of TLS past BPDE adducts. However, our results show a clear defect in cell cycle progression and cell survival following BPDE exposure in cells lacking hRev7, suggesting that cells lacking hPolζ, have trouble completing TLS (perhaps as a result of inefficient extension) past BPDE-induced DNA lesions, which ultimately results in cell death.

The crystal structure of hRev7 in complex with a fragment of hRev3 has also been recently reported [27]. In such studies the authors found that the interaction between hRev3 and hRev7 creates a structural interface that is requisite for hRev1 binding. Furthermore, they demonstrated that hRev7 mediates interactions between hRev1 and hRev3, which are critical for DNA damage tolerance. Based on their results, Hara et al. propose a mechanism whereby binding of hRev1 to the site of a fork-blocking DNA lesion functions to recruit an "inserter" polymerase to the site of DNA damage. Following the insertion step, hPolζ is recruited to the site of damage through the interaction between hRev1 and hRev7. The hRev1-hRev7 interaction also displaces the inserter polymerase from the damage site, such that hPolζ can then perform the subsequent extension step, completing TLS.

Based on data from our current study and from the studies described above, we envision that under normal circumstances, insertion opposite BPDE-induced DNA damage would be carried out by hPolκ (or hPolη). After which, hPolζ would be recruited to the DNA lesion, via interaction between hRev1 and hRev7, to perform the extension step. However, when cells lack hRev7, hPolζ would no longer be recruited to the damaged site and thus would not be capable of extending from the DNA termini. The inability to extend past the DNA lesion would result in failed TLS and, ultimately, increased cell death. Our laboratory has also recently reported that cells lacking hRev7 are more sensitive to UV-induced DNA damage and demonstrate a reduced UV-induced mutation frequency [22]. It seems likely therefore, that in the case of UV, hPolζ is required both for the insertion step (which is carried out in an error-prone manner) and for the subsequent extension step, at least for a subset of DNA lesions, for instance 6-4 photoproducts.

Finally, our data demonstrated that cell strains with reduced hRev7 are more sensitive to the cytotoxic effects of cisplatin, ionizing radiation, and MNU (Figure 4A-C), suggesting that hRev7 is required for TLS past DNA damage induced by these agents. Similar results were reported by Cheung et al. [28], who showed that downregulation of hRev7 (also referred to as MAD2B) in nasopharyngeal carcinoma cells rendered such cells more sensitive to DNA damaging agents, but not to agents whose cytotoxic effect does not generate lesions in DNA, such as anti-metabolites or microtubule-disrupting agents. The fact that the increased sensitivity of cells with reduced hRev7 is specific to DNA damaging agents is consistent with a requirement for hRev7 in TLS past DNA lesions induced by such agents.

## Conclusions

Translesion synthesis has traditionally been thought of as a rather simple process when compared with other mechanisms of DNA damage avoidance and DNA repair. However, the biological data presented here are consistent with a more complicated two-step, two-polymerase bypass mechanism requiring hPolζ or with the interpretation that there are redundant pathways for bypassing BPDE-induced DNA lesions, one of which is dependent on hPoζ and the other not requiring this polymerase. Although many aspects of TLS remain uncharacterized, studies such as these clearly indicate that TLS is much more sophisticated than first imagined and emphasize the continuing need to carry out experiments to determine the precise mechanisms involved in TLS.

## Competing interests

The authors declare that they have no competing interests.

## Authors' contributions

JAN carried out the experiments, participated in interpreting the results and in drafting of the manuscript. KLF assisted in carrying out experiments. VMM and JJM participated in designing the studies, interpreting the results and drafting the manuscript. All authors have given final approval of the version to be published.
